# Clinical Features and Antibiotic Susceptibility of *Staphylococcus aureus*-Infected Dermatoses

**DOI:** 10.3390/jcm14041084

**Published:** 2025-02-08

**Authors:** Dimitra Koumaki, Sofia Maraki, Georgios Evangelou, Vasiliki Koumaki, Stamatios Gregoriou, Stamatoula Kouloumvakou, Danae Petrou, Evangelia Rovithi, Kyriaki Zografaki, Aikaterini Doxastaki, Petros Ioannou, Ioanna Gkiaouraki, Antonios Rogdakis, Viktoria Eirini Mavromanolaki, Konstantinos Krasagakis

**Affiliations:** 1 Dermatology Department, University Hospital of Heraklion, 71110 Heraklion, Greece; gevangelou@pagni.gr (G.E.); dpetrou@pagni.gr (D.P.); eva.rovithi@pagni.gr (E.R.); kikizog@yahoo.gr (K.Z.); katerina.doxastaki@gmail.com (A.D.); igkiaouraki@pagni.gr (I.G.); arogdakis@pagni.gr (A.R.); krasagak@uoc.gr (K.K.); 2 Department of Medical Microbiology, University Hospital of Heraklion, 71110 Heraklion, Greece; smaraki@pagni.gr; 3 Department of Medical Microbiology, Medical School of Athens, National and Kapodistrian University of Athens, 75 Mikras Asias Str., Goudi, 11527 Athens, Greece; vkoumaki@med.uoa.gr; 4 Department of Dermatology and Venereology, Andreas Sygros Hospital, Medical School of Athens, National and Kapodistrian University of Athens, I. Dragoumi 5, 16121 Athens, Greece; stamgreg@med.uoa.gr; 5 2nd Department of Internal Medicine, Sismanoglio General Hospital, Sismanogliou 37, 15126 Athens, Greece; skoulou@auth.gr; 6 Department of Internal Medicine, University Hospital of Heraklion, 71500 Heraklion, Greece; p.ioannou@uoc.gr; 7 School of Medicine, University of Crete, 71003 Heraklion, Greece; 8 Department of Pediatrics, Agios Nikolaos General Hospital, 72100 Agios Nikolaos, Greece; med3548@edu.med.uoc.gr

**Keywords:** methicillin-resistant *Staphylococcus aureus*, dermatitis, skin diseases, infectious, microbial drug resistance, bacterial infections, outpatients, anti-bacterial agents

## Abstract

**Background/Objectives:** Methicillin-resistant *Staphylococcus aureus* (MRSA) poses significant treatment challenges, particularly in community settings. Limited data are available on *S. aureus*-associated infected dermatoses (ID) in outpatient dermatology clinics. This study examines the clinical characteristics, microbiological profiles, resistance patterns, and treatment outcomes of dermatoses caused by *S. aureus*. **Methods:** Between January 2023 and January 2025, consecutive patients with confirmed *S. aureus*-associated SD were recruited in a dermatology clinic in Heraklion, Greece. Demographic, clinical, and treatment data were collected. Skin swabs underwent bacterial culture and antimicrobial susceptibility testing following CLSI guidelines. Statistical analyses evaluated associations between clinical and microbiological findings. **Results:** Sixty-eight patients were included, 54.4% of whom were male, with a mean age of 46.7 years (± SD 25.1). MRSA was identified in 22.1% of cases and was significantly associated with female gender (*p* = 0.014). The most common diagnoses were eczema (35.3%) and folliculitis (19.1%). Oxacillin-resistant patients were more likely to receive systemic therapy (*p* = 0.039). Resistance rates were highest for benzylpenicillin (81.8%), levofloxacin (54.9%), and erythromycin (39.4%). Resistance rates for fusidic acid, clindamycin, mupirocin, and tetracycline were 38.2%, 20.6%, 16.9%, and 10.3%, respectively. Other pathogens, including *Pseudomonas aeruginosa* and *Escherichia coli*, were isolated in 27.9% of cases. **Conclusions:** This study highlights the high prevalence of MRSA in outpatient dermatology settings, emphasizing the need for local antimicrobial resistance surveillance to guide treatment strategies and improve outcomes in superinfected dermatoses.

## 1. Introduction

*Staphylococcus aureus* (*S. aureus*) is a major human pathogen responsible for a wide range of infections, from mild skin conditions to severe systemic diseases [1,2,3]. Methicillin-resistant *Staphylococcus aureus* (MRSA) is particularly concerning due to its resistance to beta-lactam antibiotics and its association with heightened morbidity, mortality, and healthcare costs [4,5,6]. Although traditionally linked to hospital settings, MRSA has increasingly emerged in community-acquired infections, further complicating treatment strategies. As the leading cause of skin infections, *S. aureus* presents a growing challenge with its rising antimicrobial resistance [7,8,9,10]. MRSA, in particular, has become a critical pathogen in tertiary care hospitals, where it contributes significantly to the burden of serious diseases [11,12,13,14,15,16,17]. Effective management of *S. aureus* skin infections requires a thorough understanding of antimicrobial resistance patterns, including those of both systemic and topical agents. Up-to-date surveillance data on resistance are essential to inform empirical antimicrobial selection and optimize treatment outcomes. Superinfected dermatoses (SD) with disrupted epidermis are a common presentation in dermatology clinics, particularly among patients with preexisting skin conditions, such as eczema, psoriasis, or chronic wounds [18,19,20,21,22]. These conditions disrupt the skin’s protective barrier, facilitating colonization and infection by opportunistic pathogens, including *S. aureus*. However, limited data exist on the clinical and microbiological characteristics of SD associated with *S. aureus* in outpatient dermatology settings, particularly in Mediterranean regions [15,16,17,18,19,20,21]. The increasing prevalence of antimicrobial resistance further complicates the management of these infections. MRSA’s resistance to first-line agents, coupled with the emergence of multidrug-resistant strains, underscores the importance of local surveillance to inform empiric treatment [22,23,24]. In Greece, the epidemiology of *S. aureus*-related skin infections, particularly in outpatient dermatology settings, remains poorly studied. This knowledge gap is critical given regional variations in antimicrobial resistance and clinical presentations. Understanding the clinical and microbiological profiles of *S. aureus* in SD is critical to optimizing patient care and antimicrobial stewardship. This study aims to evaluate the clinical characteristics, microbiological profiles, resistance patterns, and treatment outcomes of *S. aureus*-associated infected dermatoses (ID) in an outpatient dermatology clinic in Heraklion, Greece. By identifying local resistance trends and correlating them with clinical outcomes, this research provides valuable insights for guiding effective treatment strategies in outpatient settings.

## 2. Materials and Methods

### 2.1. Study Design and Setting

This was a prospective observational study conducted at a dermatology outpatient clinic in Heraklion, Greece, between January 2023 and January 2025. This study included consecutive patients presenting with infected dermatoses (ID) characterized by disrupted epidermis and confirmed *Staphylococcus aureus* (*S. aureus*) infection.

### 2.2. Patient Selection

Patients were included if they met the following criteria: (1) clinical evidence of SD, including erythema, pustules, or exudate; (2) microbiological confirmation of *S. aureus* from skin swabs; and (3) age ≥18 years. Patients were excluded if they had received systemic antibiotics within 30 days prior to sampling or had other systemic infections. Written informed consent was obtained from all participants.

### 2.3. Sample Collection, Transport, and Processing

To prevent contamination, the skin was cleansed with a solution of 2% chlorhexidine gluconate and 70% isopropyl alcohol before sample collection. Pus and exudates were collected from affected areas using sterile cotton swabs and placed in an Amies transport medium (bioMérieux SA, Marcy l’Etoile, France). Samples were transported to the Microbiology Laboratory via a pneumatic tube system and processed within 30 min. Standardized procedures included wet mount preparation, Gram staining, and culturing on Columbia agar with 5% sheep blood, chocolate agar with polyvitex, mannitol salt agar, Drigalski lactose agar, and Sabouraud agar under aerobic conditions at 36 °C. Shaedler agar with 5% sheep blood was used for anaerobic cultures. The Vitek 2 automated system and MALDI-TOF MS (version 3.2, bioMérieux SA) were used for bacterial identification. Antibiotic susceptibility testing followed the 2023 Clinical and Laboratory Standards Institute (CLSI) guidelines, with reference strains (*Escherichia coli* ATCC 25922, *Pseudomonas aeruginosa* ATCC 27853, *S. aureus* ATCC 25923, and *Enterococcus faecalis* ATCC 29212) employed for quality control [22,23].

### 2.4. Statistical Analysis

Data were analyzed using IBM SPSS Statistics (Version 25.0). Completeness and consistency of the dataset were ensured prior to analysis. Categorical variables were analyzed for frequency distributions, while continuous variables underwent exploratory data analysis to identify anomalies. Missing data were excluded from relevant analyses. All analysis steps were documented to ensure transparency and reproducibility.

### 2.5. Ethical Considerations

This study was reviewed and approved by the Ethical Committee of the University Hospital of Heraklion, Heraklion, Greece, on 18 October 2022, with approval number 22223/18-10-2022.

## 3. Results

A total of 68 consecutive adult patients, from whom *S. aureus* was isolated from skin swabs from areas of superinfected dermatoses (SD) with disrupted epidermis, were recruited between January 2023 and January 2025. The cohort included 54.4% (37/68) males and 45.6% (31/68) females, with a mean age of 46.71 years (± SD 25.05). MRSA was isolated in 15/68 (22.1%) samples, while MSSA was detected in 53/68 (77.9%). MRSA was significantly associated with female gender (73.3% females; *p* = 0.014). Among patients with *S. aureus*, 35.3% (24/68) had eczema, 19.1% (13/68) had folliculitis, 10.3% (7/68) had hidradenitis suppurativa, and 7.4% (5/68) had psoriasis (Table 1). Before the collection of samples, the topical treatments used by patients included emollients (42/68, 61.76%), topical corticosteroids (38/68, 55.88%), topical adapalene (8/68, 11.76%), and calcitriol topical ointment (5/68, 7.4%). Of the 68 patients, 11 (16.2%) were immunosuppressed. The immunosuppressive drugs taken by these 11 patients included oral prednisolone (5/11, 45.4%), oral methotrexate (3/11, 27.3%), azathioprine (2/11, 18.2%), and mycophenolate mofetil (MMF) (1/11, 9.1%).

Of the *S. aureus* isolates, 28/68 (41.2%) were from the lower limbs, 13/68 (19.1%) from the upper limbs, and 10/68 (14.7%) from the trunk. Additionally, 11/68 (16.2%) of the patients were immunosuppressed, and all 68/68 (100%) *S. aureus* isolates were community-acquired. Topical treatments were administered to 52.9% (36/68) of the patients, while 47.1% (32/68) received systemic therapy. Treatments included fusidic acid (27.9%), topical mupirocin (16.2%), topical clindamycin (8.8%), oral clindamycin (14.7%), oral cefuroxime axetil (13.2%), oral erythromycin (10.3%), and oral trimethoprim/sulfamethoxazole (1.5%). All MRSA patients were treated systemically (*p* = 0.039). Other bacteria were isolated in 27.9% (19/68) of the patients, including *Acinetobacter baumannii*, *Achromobacter denitrificans*, *Candida albicans*, *Corynebacterium simulans*, *Enterobacter cloacae ssp cloacae*, *Escherichia coli*, *Κlebsiella aerogenes*, *Proteus mirabilis*, *Pseudomonas aeruginosa*, *Serratia marcescens*, *Staphylococcus epidermidis*, *Staphylococcus lugdunensis, Staphylococcus pyogenes*, *and Streptococcus mitis/Streptococcus oralis* (Table 2).

Antibiotic resistance rates were highest for benzylpenicillin (81.8%), followed by levofloxacin (54.9%), erythromycin (39.4%), fusidic acid (38.2%), oxacillin (22.1%), clindamycin (20.6%), mupirocin (16.9%), gentamicin (10.3%), and tetracycline (10.3%) (Table 3).

## 4. Discussion

This study evaluates the clinical and microbiological features of infected dermatoses (ID) caused by *Staphylococcus aureus* (*S. aureus*) in a dermatology outpatient setting in Greece, providing insights into community-acquired infections and their resistance patterns. Notably, 22.1% of *S. aureus* isolates were methicillin-resistant (MRSA), aligning with rates in Southern Europe, where antibiotic overuse has driven resistance [22]. In contrast, Northern Europe shows lower MRSA rates due to stricter antibiotic stewardship. Community-acquired methicillin-resistant *Staphylococcus aureus* (CA-MRSA) is increasingly recognized as a significant cause of skin and soft tissue infections, particularly among otherwise healthy individuals. Studies indicate that CA-MRSA accounts for approximately 30.2% of MRSA infections in various populations, with some regions reporting even higher prevalence rates, such as 64% among patients with skin infections in emergency departments. The predominant clinical manifestations of CA-MRSA include skin abscesses and folliculitis, often found in young athletes and individuals engaged in close-contact sports Furthermore, a meta-analysis revealed that the prevalence of CA-MRSA among skin and soft tissue infections can vary widely, with rates ranging from 1.9% to 96%, highlighting the need for ongoing surveillance and tailored treatment strategies. Environmental factors, such as crowded living conditions, shared personal items, and direct skin-to-skin contact have been identified as key contributors to the spread of CA-MRSA in community settings [24,25,26]. Overall, while CA-MRSA remains a critical public health concern, understanding its epidemiology is essential for effective prevention and management strategies.

The study also found a significant association between MRSA and female gender (73.3%, *p* = 0.014), which is in contrast with findings of recent literature warranting further investigation into potential contributing factors. Recent literature supports the significant association between female gender and the prevalence of community-acquired methicillin-resistant *Staphylococcus aureus* (CA-MRSA) infections in skin conditions [27]. A study found that females exhibited a lower incidence of *Staphylococcus aureus* skin and soft tissue infections (SSTIs) compared to males, with an odds ratio of 2.4 for male infections, indicating a pronounced male predominance in these cases. Additionally, gender differences in susceptibility to CA-MRSA have been attributed to biological factors, such as the protective effects of estrogen, which have been shown to enhance immune responses and reduce inflammation during infections [28]. Furthermore, research indicates that while males are more frequently colonized by MRSA, females may experience more severe outcomes from bloodstream infections, highlighting complex interactions between gender and infection dynamics. These findings suggest that gender-specific factors play a crucial role in the epidemiology of MRSA infections, warranting further investigation into tailored prevention and treatment strategies.

Eczema (35.3%) and folliculitis (19.1%) were the most common diagnoses, reflecting *S. aureus*’s role in inflammatory skin conditions.

Resistance to benzylpenicillin in *Staphylococcus aureus* was high, at a value of 81.8%, which aligns with previously published findings showing near-universal prevalence due to widespread beta-lactamase production [24]. Historical data indicate that benzylpenicillin resistance in *Staphylococcus aureus* began emerging in the late 1940s, shortly after penicillin’s introduction, and by the 1960s, over 80% of clinical *S. aureus* isolates globally were resistant [25,26]. Modern surveillance studies confirm that resistance rates remain consistently high in both community and hospital settings, often exceeding 90%, particularly in regions where penicillin use is uncontrolled [4,5]. The primary driver of resistance is the blaZ gene, which encodes beta-lactamase and is often carried on plasmids, facilitating its rapid dissemination among bacterial populations. Due to this near-universal resistance, benzylpenicillin is rarely used in treating *S. aureus* infections, with beta-lactamase-stable antibiotics or non-beta-lactam agents being preferred alternatives [8,9,10,11,12].

Resistance to fusidic acid (38.2%), erythromycin (39.4%), and mupirocin (16.9%) highlights the need for cautious use of topical antibiotics.

In our study, the resistance rate (38.2%) of fusidic acid in *S. aureus* isolates was relatively high according to other published studies.

Resistance to fusidic acid in *Staphylococcus aureus* has been increasingly reported in community settings, with rates varying widely depending on geographic location and antibiotic usage patterns. Fusidic acid resistance typically arises through mutations in the fusA gene, encoding elongation factor G, or through the acquisition of resistance genes such as *fusB* or *fusC*, which are often plasmid-mediated [29,30,31,32,33,34,35]. A recent systematic review and meta-analysis on the global prevalence of fusidic acid resistance in clinical isolates of *Staphylococcus aureus* revealed that the overall prevalence rates of fusidic-acid-resistant *S. aureus* (FRSA), fusidic-acid-resistant MRSA (FRMRSA), and fusidic-acid-sensitive *S. aureus* (FRMSSA) were 0.5%, 2.6%, and 6.7%, respectively [35,36].

Our study identified a 16.9% resistance rate to mupirocin in *Staphylococcus aureus* samples from superinfected dermatoses, which is notably higher than the resistance rates reported in most global studies. A comprehensive global review revealed pooled mupirocin resistance rates of 7.6% (95% CI: 6.2–9.0%) for mupirocin-resistant *S. aureus* (MuRSA), highlighting regional variations and a rising trend in resistance development [37,38]. Studies from India have reported high-level mupirocin resistance at 11.93%, suggesting local factors, such as mupirocin overuse or misuse, may play a significant role in driving resistance [39,40,41,42,43,44]. Another investigation found mupirocin resistance rates reaching 31.3% among all *S. aureus* isolates, particularly in healthcare settings where mupirocin is frequently used for decolonization, further supporting the link between prior mupirocin exposure and resistance emergence [45]. High-level mupirocin resistance has also been associated with the presence of the *mupA* gene, often located on mobile genetic elements such as plasmids that may carry additional resistance genes, contributing to multidrug resistance [46]. Furthermore, certain studies have indicated that the use of mupirocin for nasal decolonization in hospital environments might accelerate resistance development, raising concerns about its continued efficacy as a preventive measure. These findings underscore the critical need for surveillance of mupirocin resistance, judicious use of the antibiotic, and exploration of alternative therapies to manage *S. aureus* infections effectively.

In our study, the prevalence of inducible clindamycin resistance among MRSA isolates was 10.9%, which aligns with findings reported in the literature. A recent systematic review estimated the prevalence of inducible clindamycin resistance in *S. aureus* isolates to range from 2.9% to 44% [47,48]. The highest documented prevalence was 44.0%, reported in Egypt in 2017, while the lowest, 2.9%, was observed in Côte d’Ivoire [49]. Additional peaks of 35.8% and 33.3% were recorded in 2007 and 2013, respectively. In central Greece, clindamycin resistance among CA-MRSA isolates in pediatric clinical samples rose significantly from 0% in 2003 to 31.2% in 2009 (*p* = 0.011). The increased use of clindamycin, particularly for pediatric infections, has been attributed to its effectiveness against a high percentage of CA-MRSA and MSSA isolates [50,51,52,53,54,55]. In regions with low clindamycin resistance, this antibiotic remains a recommended empirical therapy for hospitalized pediatric patients with likely CA-MRSA infections, such as cutaneous abscesses or pneumonia with empyema [56].

In our study, we identified a tetracycline resistance rate of 10.3% among MRSA isolates in superinfected dermatoses, a figure consistent with prior research but indicative of the growing threat of antimicrobial resistance. The prevalence of tetracycline resistance in MRSA skin infections has been reported to vary widely. One study revealed a resistance rate of 16%, suggesting that tetracyclines could remain effective in certain MRSA infections [57]. However, longitudinal analyses have demonstrated a troubling increase in resistance, with rates climbing from 3.6% in 2010 to 12.8% in 2019, reflecting an upward trend in resistance [57]. Alarmingly, resistance rates as high as 60.6% have been documented, attributed to the dissemination of tetracycline-resistant genes, such as *tetK* and *tetM*, which are often plasmid-mediated and transferable between bacterial strains [58]. Regional variability further complicates this issue, with some areas reporting resistance rates exceeding 50%, particularly in regions with higher antibiotic consumption or less stringent antimicrobial stewardship programs. These findings highlight the importance of ongoing surveillance of resistance patterns and the judicious use of tetracyclines to preserve their efficacy. Future research should focus on identifying mechanisms driving resistance and developing alternative therapeutic options.

In our study, we observed a 54.9% resistance rate to levofloxacin in MRSA isolates from superinfected dermatoses, underscoring the growing challenge of antimicrobial resistance in dermatological settings. Resistance to levofloxacin among MRSA in superinfected skin conditions has become an increasing concern. Previous research reported a 53.5% resistance rate in MRSA strains isolated from hand infections, highlighting the pervasive nature of this resistance pattern in localized infections [58,59]. A longitudinal analysis from 2016 to 2021 demonstrated an increase in levofloxacin resistance among MRSA isolates across different sources, rising from 5.45% to 7.14%, suggesting an incremental but significant trend [60,61]. Additionally, studies on invasive MRSA infections have shown even greater disparities, with blood-derived MRSA strains exhibiting resistance rates as high as 15.38% compared to those from soft tissue sources. Geographic variability in resistance rates has also been noted, with studies from Egypt documenting alarmingly high rates of up to 78%, particularly among specific MRSA clones, further underscoring the role of regional factors and genetic variations in shaping resistance trends. These findings emphasize the urgent need for continuous antimicrobial resistance surveillance and tailored antibiotic stewardship programs to mitigate the threat posed by levofloxacin-resistant MRSA. Future research should also explore the underlying mechanisms driving resistance and evaluate alternative therapeutic options to optimize treatment outcomes for MRSA-associated infections.

Despite resistance concerns, rifampicin and vancomycin remain effective against MRSA. All MRSA patients required systemic therapy (*p* = 0.039), underscoring the limitations of topical treatments due to biofilm formation and deeper tissue involvement. Polymicrobial infections, found in 27.9% of cases, further complicate treatment, with pathogens like *Pseudomonas aeruginosa* requiring targeted therapy. While MRSA prevalence in this study exceeded 10%, empirical MRSA therapy for skin infections is not recommended in this context. This study emphasizes the importance of tailored treatment strategies based on local epidemiology.

Our study provides insights into the clinical features and antibiotic susceptibility of *Staphylococcus aureus*-infected dermatoses. However, it is important to recognize that *S. aureus* infections can present with a broader spectrum of clinical manifestations, including more severe systemic and dermatologic conditions. Among these, staphylococcal scalded skin syndrome (SSSS), toxic shock syndrome (TSS), and staphylococcal scarlatiniform eruption represent critical dermatologic and systemic entities [62,63,64,65,66,67,68].

SSSS is caused by exfoliative toxins produced by certain strains of *S. aureus*, targeting desmoglein-1 and leading to widespread epidermal detachment. Clinically, SSSS begins with fever, irritability, and diffuse erythema, progressing to superficial blistering and exfoliation, particularly in neonates and immunocompromised individuals. A distinguishing feature of SSSS is its histological presentation, which reveals intraepidermal cleavage without significant dermal inflammation [62,63,64]. Given its potential severity, early recognition and appropriate antibiotic therapy are crucial.

TSS is a life-threatening condition resulting from superantigenic toxins such as toxic shock syndrome toxin-1 (TSST-1) and enterotoxins, triggering an excessive immune response leading to fever, hypotension, and multiorgan dysfunction. Dermatologic findings include a diffuse erythematous rash followed by desquamation, particularly on the palms and soles [67]. TSS has been associated with tampon use, surgical wounds, and other invasive *S. aureus* infections. The rapid progression and systemic involvement necessitate urgent medical intervention, including aggressive supportive care and targeted antimicrobial therapy.

Another underrecognized manifestation is staphylococcal scarlatiniform eruption, presenting as a febrile illness with a generalized erythematous rash resembling scarlet fever [68]. Often associated with early-stage SSSS, it is characterized by diffuse erythema and tenderness, particularly in flexural areas. Unlike streptococcal scarlatiniform eruptions, which exhibit a classic “sandpaper-like” texture, the staphylococcal variant is more tender and may progress to exfoliation. Awareness of this entity can aid in distinguishing it from other exanthematous conditions.

While purpura fulminans is more commonly linked to *Neisseria meningitidis*, there are reports of its association with TSST-1-producing *S. aureus*. This rare but severe manifestation presents with widespread purpuric and necrotic lesions due to disseminated intravascular coagulation. A reported case highlights the aggressive nature of staphylococcal purpura fulminans, demonstrating its rapid progression to multiorgan failure and high mortality rates. The recognition of such severe presentations underscores the importance of early intervention and appropriate antimicrobial therapy.

The diverse clinical spectrum of *S. aureus* infections extends beyond conventional dermatoses to include severe dermatologic and systemic manifestations. Recognizing conditions such as SSSS, TSS, staphylococcal scarlatiniform eruption, and purpura fulminans is crucial for timely diagnosis and management. Future research should focus on the epidemiology and pathogenesis of these severe manifestations, as well as strategies to optimize treatment outcomes. Our study highlights the importance of continued surveillance of antibiotic susceptibility patterns to ensure effective management of both common and severe *S. aureus* infections.

This study has several strengths, including its comprehensive evaluation of clinical and microbiological features of *Staphylococcus aureus*-associated superinfected dermatoses (SD) in a community setting, focusing on both MRSA and MSSA strains. By addressing the underexplored issue of community-acquired infections in outpatient dermatology clinics in the Mediterranean region, it provides valuable data on local antimicrobial resistance patterns, particularly highlighting high resistance rates to levofloxacin (54.9%) and mupirocin (16.9%). The use of rigorous microbiological techniques, adherence to CLSI guidelines, and the inclusion of diverse dermatological conditions strengthen the reliability and applicability of the findings.

However, this study is limited by its relatively small sample size and geographic scope, which may reduce generalizability to other regions. Additionally, its cross-sectional design precludes analysis of temporal resistance trends, and the exclusion of pediatric patients limits its applicability to younger populations. Furthermore, this study did not account for prior topical antibiotic use, which could influence resistance patterns, particularly for mupirocin or fusidic acid. Despite these limitations, this study provides essential insights into local resistance trends, aiding the development of targeted treatment strategies.

## 5. Conclusions

This study provides valuable insights into the clinical and microbiological profiles of superinfected dermatoses (SDs) caused by *Staphylococcus aureus* in a dermatology outpatient clinic in Heraklion, Greece. The findings highlight significant resistance rates to commonly used antibiotics, including levofloxacin (54.9%) and mupirocin (16.9%), underscoring the critical need for local surveillance to inform empirical treatment strategies. Additionally, the significant association of MRSA with female gender raises intriguing questions for future research. While this study emphasizes the growing challenge of managing community-acquired *S. aureus* infections amidst rising resistance, it also underscores the importance of individualized treatment approaches and judicious antibiotic use to mitigate further resistance development. Despite some limitations, this research contributes to a deeper understanding of *S. aureus*-associated SD and serves as a foundation for improving patient care and antimicrobial stewardship in outpatient dermatology settings.

## Figures and Tables

**Table 1:** Clinical features of 68 patients with *Staphylococcus aureus* identified in skin samples, and statistical correlations with MRSA status. A *p*-value of <0.05 was considered statistically significant.

Variable		*p*-Value
MRSA status		
MRSA	15/68 (22.1%)	
MSSA	53/68 (77.9%)	
Gender		*p* = 0.014
Male N, %	37/68 (54.4%)	
Males with MRSA	4/15 (26.7%)	
Males with MSSA	33/53 (62.3%)	
Female N, %	31/68 (45.6%)	
Females with MRSA	11/15 (73.3%)	
Females with MSSA	20/53 (37.7%)	
Age, years Mean	46.71	*p* = 0.155
Median ± SD	42 ± 25.05	
Age groups		*p* = 0.141
From 0- to 18-year-olds	7/68 (10.3%)	
Above the age of 66	61/68 (89.7%)	
Sample type		*p* = 0.303
Exudate	42/68 (61.8%)	
Pus	26/68 (38.2%)	
Sample site		*p* = 0.257
Head and neck	9/68 (13.2%)	
Upper limbs	13/68 (19.1%)	
Trunk	10/68 (14.7%)	
Buttocks	6/68 (8.8%)	
Inguinal fold/genital area	2/68 (2.9%)	
Lower limbs	28/68 (41.2%)	
Body site		*p* = 0.542
Above waist	32/68 (47.1%)	
Below waist	36/68 (52.9%)	
Trauma history, N %	13/68 (19.1%)	*p* = 0.407
Clinical presentation		*p* = 0.849
Eczema	24/68 (35.3%)	
Folliculitis	13/68 (19.1%)	
Hidradenitis suppurativa (HS)	7/68 (10.3%)	
Psoriasis	5/68 (7.4%)	
Impetigo	3/68 (4.4%)	
Rosacea	3/68 (4.4%)	
Paronychia	3/68 (4.4%)	
Lichen planus	2/68 (2.9%)	
Bullous pemphigoid	2/68 (2.9%)	
Lichen sclerosus	1/68 (1.5%)	
Darier’s disease	1/68 (1.5%)	
Pemphigus	1/68 (1.5%)	
Mycosis fungoides	1/68 (1.5%)	
Grover’s disease	1/68 (1.5%)	
Kaposi sarcoma	1/68 (1.5%)	
Other bacteria isolated		*p* = 0.903
No other bacteria were isolated	49/68 (72.1%)	
Yes, other bacteria were also isolated	19/68 (27.9%)	
Hospital-acquired		
No	0/68 (0%)	
Community-acquired		
Yes	68/68 (100%)	
Immunological status		*p* = 0.655
Immunosuppressed	11/68 (16.2%)	
Immunocompetent	57/68 (83.8%)	
Immunosuppressed medications used		*p* = 0.768
Oral prednisolone	5/11 (45.4%)	
Oral methotrexate (MTX)	3/11 (27.3%)	
Azathioprine (AZTH)	2/11 (18.2%)	
Mycophenolate mofetil (MMF)	1/11 (9.1%)	
Other topical treatments used before recruitment to the study		*p* = 0.864
Emollients	42/68 (61.76%)	
Topical corticosteroids	38/68 (55.88%)	
Topical adapalene	8/68 (11.76%)	
Topical calcitriol ointment	5/68 (7.4%)	
Treatment		*p* = 0.087
Topical treatment	36/68 (52.9%)	
Systemic antibiotic treatment	32/68 (47.1%)	
Type of treatment		*p* = 0.039
Topical fusidic acid	19/68 (27.9%)	
Topical mupirocin	11/68 (16.2%)	
Topical clindamycin	6/68 (8.8%)	
Oral doxycycline	10/68 (14.7%)	
Oral amoxicillin/clavulanic acid	9/68 (13.2%)	
Oral cefuroxime axetil	7/68 (10.3%)	
Oral erythromycin	5/68 (7.4%)	
Oral trimethoprim/sulfamethoxazole	1/68 (1.5%)	
Treatment outcome		
Cure	68/68 (100%)	

**Table 2:** Other bacteria and fungi isolated from the 68 skin specimens positive for *Staphylococcus aureus*.

*Escherichia coli*
*Κlebsiella aerogenes*
*Proteus mirabilis* *Enterobacter cloacae*
*Serratia marcescens* *Pseudomonas aeruginosa*
*Acinetobacter baumannii* *Achromobacter denitrificans*
*Staphylococcus epidermidis*
*Staphylococcus lugdunensis*
*Streptococcus pyogenes*
*Streptococcus mitis/oralis*

**Table 3:** Antibiotic resistance profiles of the 68 *Staphylococcus aureus* isolates from skin specimens.

Antimicrobial Agents	Susceptible N (%)	Resistant N (%)
Benzylpenicillin	12/66 (18.2%)	54/66 (81.8%)
Oxacillin	53/68 (77.9%)	15/68 (22.1%)
Gentamicin	61/68 (89.7%)	7/68 (10.3%)
Levofloxacin	23/51 (45.1%)	28/51 (54.9%)
Erythromycin	40/66 (60.6%)	26/66 (39.4%)
Clindamycin	54/68 (79.4%)	14/68 (20.6%)
Linezolid	68/68 (100%)	0/68 (0%)
Daptomycin	62/62 (100%)	0/62 (0%)
Teicoplanin	62/68 (91.2%)	6/68 (8.8%)
Vancomycin	68/68 (100%)	0/68 (0%)
Tetracycline	61/68 (89.7%)	7/68 (10.3%)
Tigecycline	68/68 (100%)	0/68 (0%)
Fusidic acid	42/68 (61.8%)	26/68 (38.2%)
Mupirocin	54/65 (83.1%)	11/65 (16.9%)
Rifampicin	68/68 (100%)	0/68 (0%)
Trimethoprim/sulfamethoxazole	65/68 (95.6%)	3/68 (4.4%)

## Data Availability

The data that support the findings of this study are available from the corresponding author upon reasonable request.

## Abbreviations

The following abbreviations are used in this manuscript:

MRSA	methicillin-resistant *Staphylococcus aureus*
*S. aureus*	*Staphylococcus aureus*
CLSI	Clinical and Laboratory Standards Institute
ID	infected dermatoses
MuRSA	mupirocin-resistant *S. aureus*
SSSS	staphylococcal scalded skin syndrome
TSS	toxic shock syndrome
